# Examination of Macular Retina and Choroidal Thickness in High Myopic Amblyopia Using Spectral-Domain Optical Coherence Tomography

**DOI:** 10.3389/fmed.2022.808409

**Published:** 2022-03-28

**Authors:** Juan Wan, Zhengwei Zhang, Yu Tian

**Affiliations:** ^1^Department of Ophthalmology, Guangzhou Hospital of Integrated Traditional and Western Medicine, Guangzhou, China; ^2^Department of Ophthalmology, Affiliated Wuxi Clinical College of Nantong University, Wuxi, China; ^3^Department of Ophthalmology, The Affiliated Wuxi No. 2 People's Hospital of Nanjing Medical University, Wuxi, China; ^4^Department of Ophthalmology, Hunan Children's Hospital, Changsha, China

**Keywords:** myopia, amblyopia, retina thickness, choroid thickness, OCT

## Abstract

**Purpose:**

The aim of this study was to investigate changes in the retinal and choroidal thickness between high myopic amblyopia (HMA), low myopia (LM), moderate myopia (MM), high myopia (HM), and normal group (NG) using a spectral-domain optical coherence tomography (SD-OCT).

**Materials and Methods:**

A total of 75 Chinese children (128 eyes; mean age 10.5 years) were recruited. Retinal thickness (RT) and choroidal thickness (CT) were measured at different locations including subfoveal (SF), and at 0.5 mm/1.0 mm/1.5 mm/2.0 mm/2.5 mm/3.0 mm to the fovea in superior, nasal, inferior, and temporal sectors using enhanced depth imaging (EDI) system of SD-OCT. Axial length (AL), best-corrected visual acuity (BCVA), and refraction errors were also collected.

**Results:**

No significant differences were found in subfoveal retinal thickness (SFRT). Moreover, a significantly thinner subfoveal choroidal thickness (SFCT) was found in HMA compared to NG, LM, and MM, but not compared to HM. RT at 0.5 mm to fovea, HMA was significantly thinner compared to LM and MM in the three sectors (superior, inferior, and temporal). Nevertheless, no significant differences were found compared to NG and HM. CT at 0.5 mm to fovea, HMA was the significantly thinnest in all four sectors compared to NG, LM, and MM. RT at 1.0 mm/1.5 mm/2.0 mm/2.5 mm/3.0 mm to fovea, HMA was thinner compared to NG, LM, and MM. CT at 1.0 mm/1.5 mm/2.0 mm/2.5 mm/3.0 mm to fovea, HMA was thinner compared to NG, LM, and MM. At the superior and inferior sectors, HMA showed to be statistically thinner compared with HM. Moreover, SFCT in the HMA, HM, and NG were negatively correlated with AL.

**Conclusions:**

Thinner retina and choroidal tissue appear to be related to HMA, and thus can be used as useful parameters for discovering the underlying mechanisms of the disease.

## Introduction

Amblyopia is a neuro-developmental disorder of the visual cortex, caused by visual deprivation or abnormal binocular interactions, including unilateral strabismus, no corrective anisometropia, and high refractive error (1). Amblyopia is the most common cause of vision loss in children and adolescents, with an estimated incidence of 1–3.5% worldwide (2). Early detection and treatment offer the best outcome. If not detected and treated early in life, amblyopia can cause a permanent loss of vision (3). Compared to unilateral amblyopia, such as anisometropic amblyopia and strabismic amblyopia that offer good treatment outcomes especially at younger age (4), refractive correction of amblyopia combined with high myopia (HM) is a difficult clinical problem. Currently, there is no satisfactory surgical procedure for the correction of high myopic amblyopia (HMA).

It has been reported that the pathogenesis of amblyopia may involve various levels of visual pathways, such as the visual cortex, lateral geniculate nucleus, retina, and optic nerve (5, 6). Meanwhile, retinal abnormalities mainly include retinal photoreceptor cells, retinal pigment epithelium (RPE), retinal nerve fiber layer, and optic nerve abnormalities (5–7). Choroid is an important tissue structure in the eyeball, located in the outer layer of the retina, which provides nutrients and oxygen to the retinal pigment cells and the optic nerve (8). Previous studies have suggested that changes in retinal and choroidal thickness may be involved in amblyopia. However, the results are disputed (9, 10).

With the development of optical coherence tomography (OCT) technology, it has become possible to measure and analyze the choroidal thickness (CT) (11). Over the recent years, many studies have investigated the changes in CT in various eye diseases. Pachychoroid is a relatively new concept describing a phenotype characterized by attenuation of the choriocapillaris overlying dilated choroidal veins, and progressive retinal pigment epithelium dysfunction and neovascularization are also present (12). Pachychoroid disease spectrum includes (12–14) central serous chorioretinopathy (CSC) (12–15), pachychoroid neovasculopathy (PNV) (12–14), peripapillary pachychoroid syndrome (PPS) (12–14), polypoidal choroidal vasculopathy (PCV) (12–14, 16) /aneurysmal type 1 neovascularization (AT1) (12–14), focal choroidal excavation (FCE) (12–14), and pachychoroid pigment epitheliopathy (PPE) (12–14, 17). These images and data suggest that choroid has an important role in the occurrence and development of ophthalmic diseases. Similarly, choroid is closely related to axial length (AL) and refractive error. Myopia is associated with a higher spherical equivalent (SE), a longer AL, and thinner CT (18). Teberik et al. (19) have reported that subfoveal choroidal thickness (SFCT) was significantly thinner in the HM group than the healthy subjects (*p* < 0.001). However, according to our knowledge, there are no studies that have examined the retinal thickness (RT) and CT in HMA.

The aim of this study was to investigate changes in the retina and choroid from among HMA, low myopia (LM), moderate myopia (MM), HM, and normal group (NG) using spectral-domain optical coherence tomography (SD-OCT).

## Materials and Methods

### Ethics Statement

This study was approved by the local ethics committee of Guangzhou hospital of integrated traditional and western medicine and was in accordance with the principles of the Declaration of Helsinki. All subjects and their guardians provided written informed consent.

### Study Population and Data Collection

A total of 75 Chinese children (128 eyes) were recruited from the department of ophthalmology at Guangzhou hospital of integrated traditional and western medicine, between July 2014 and November 2014 (Table 1). The subjects were aged between 4 and 15 years. All participants underwent a comprehensive ophthalmic examination, including visual acuity (VA), slit-lamp biomicroscopy, cycloplegic refraction, dilated fundus examination with indirect ophthalmoscopy, and A-scan ultrasound biometry for measuring AL. In addition, each participant had no history of other ocular diseases, and a detailed medical history was also obtained.

**Table 1:** Demographic and clinical data for each group.

	**Normal group**	**Low myopia group**	**Moderate myopia group**	**High myopia group**	**High myopia amblyopia group**
Eyes	22	37	22	22	25
Age	10.18 ± 2.63	9.97 ± 2.30	12.09 ± 2.30	13.11 ± 2.35	7.13 ± 2.27
Male:female	11:8	8:5	7:6	7:6	14:3
VA/BCVA	1.01 ± 0.11	0.97 ± 0.06	0.95 ± 0.08	0.95 ± 0.09	0.33 ± 0.16
Axial length (mm)	23.04 ± 0.89	24.24 ± 0.71	25.02 ± 0.92	26.14 ± 1.03	26.70 ± 1.32

Visual acuity was measured using LogMAR E chart, and refractive (spherical equivalent, SE) error was determined using retinoscopy with cyclopentolate 1%. All subjects had astigmatism ≤1.0 D without strabismus. For NG (22 eyes), VA should be ≥0.6 for children aged 4 years, ≥0.8 for children aged 5–6 years, and 1.0 or better for children aged 7 years and above. The SE was between −0.5 D and −3.0 D for LM, between −3.0 D and −6.0 D for MM, and more than 6.0 D for HM. Amblyopia is usually classified as BCVA should be < 0.5 for children aged 4, < 0.6 for children aged 5–6 < 0.8 for children aged 7 and above, and an interocular difference in SE of less than 2.0 D in the same child.

**Figure 1:**
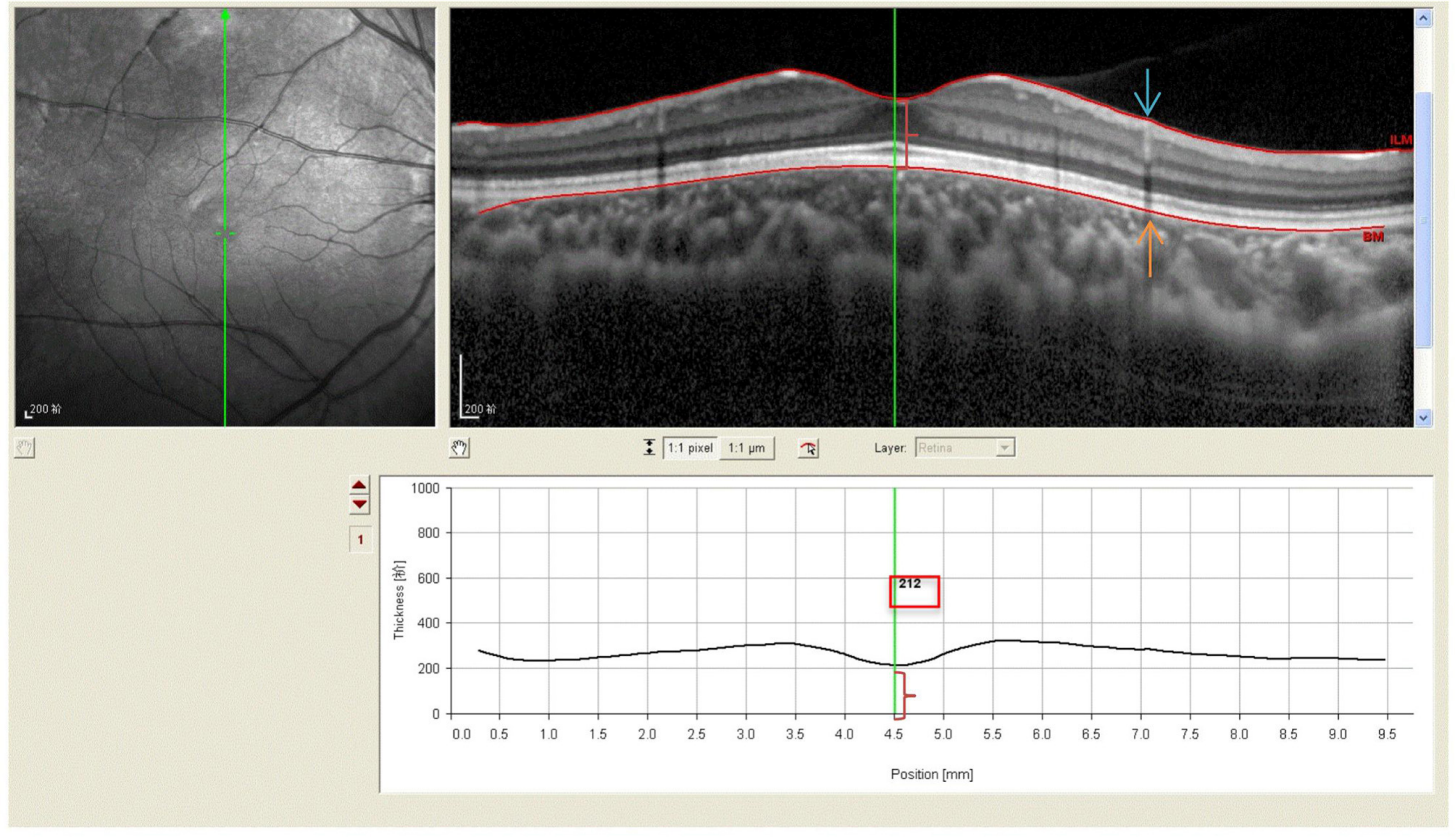
Spectral-domain optical coherence tomography (SD-OCT) scans showing retinal thickness of high myopic amblyopic eyes. (Top left) Fundus image showing vertical scan lines go through the fovea in 6-year-old subject. (Top right) The retinal thickness (red brace) was defined from ILM (redline, blue arrow) to the basal aspect of the RPE (redline, yellow arrow). (Bottom) The retinal thickness 212 μm (red brace, red boxes) was measured vertically at the subfoveal by Heidelberg own software, and at 500 μm intervals from the fovea to 3 mm superior, inferior.

**Figure 2:**
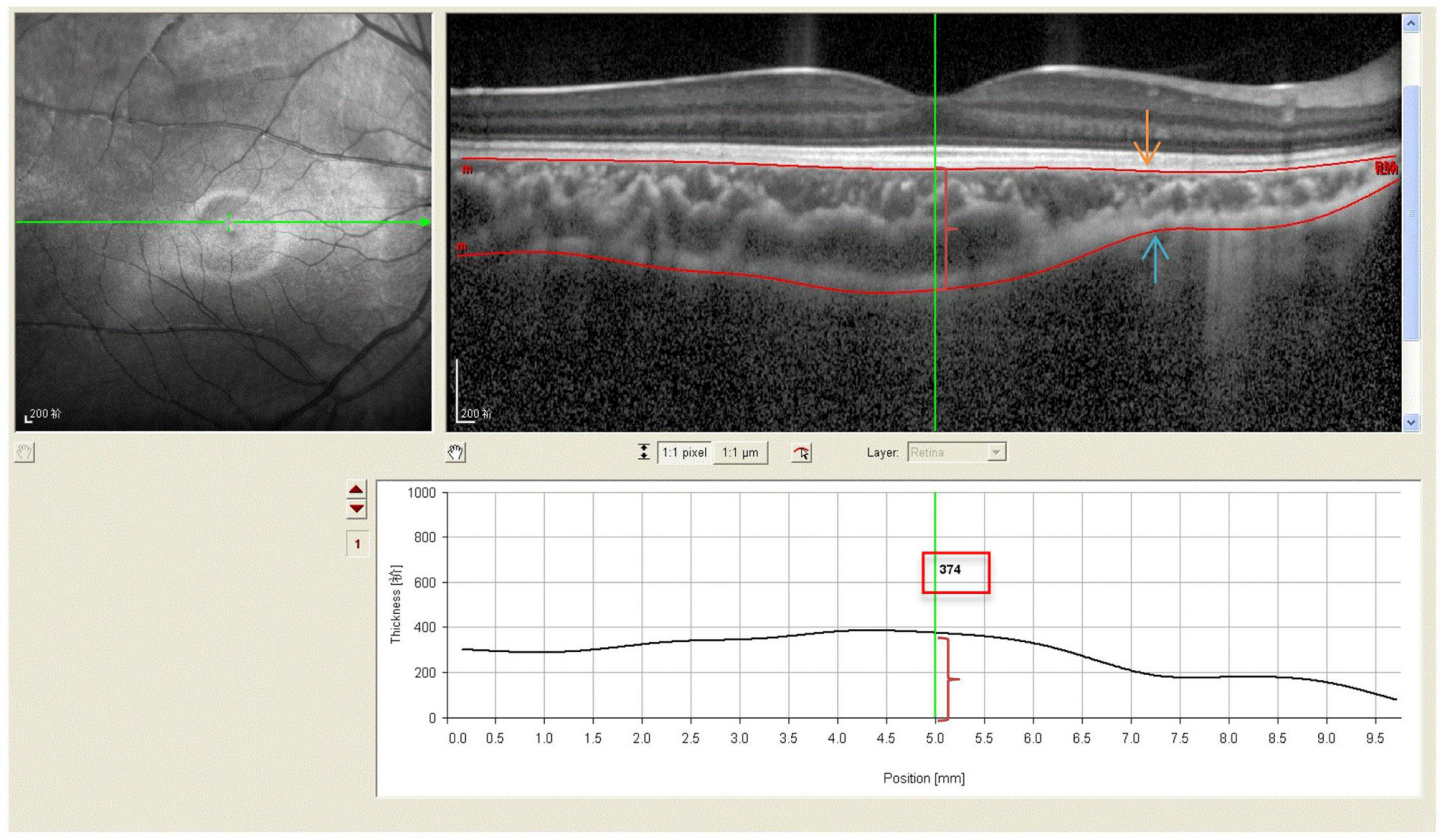
Spectral-domain optical coherence tomography (SD-OCT) scans showing choroidal thickness of high myopic amblyopic eyes. (Top left) Fundus image showing horizontal scan lines go through the fovea in 6-year-old subject. (Top right) The choroidal thickness (red brace) was defined from the basal aspect of the RPE (redline,yellow arrow) to the outer border of the choroid (redline, blue arrow). (Bottom) The choroidal thickness 374 μm (red brace, red boxes) was measured vertically at the subfoveal by Heidelberg own software, and at 500 μm intervals from the fovea to 3 mm nasal, temporal.

All subjects with dilated pupils were examined using enhanced depth imaging (EDI) system of SD-OCT (Heidelberg Engineering, Heidelberg, Germany). All imaging data were collected by a single skilled technician during the day, but not at the same time due to randomness of the subjects' visits. Each image was averaged for 100 scans using the automatic averaging and eye tracking characteristics. The RT was measured from the outer portion of the hyperreflective line corresponding to the internal limiting membrane (ILM) to the retinal pigment epithelium (RPE) (Figure 1). CT was measured from the outer portion of the hyperreflective line corresponding to RPE to the inner surface of the sclera (Figure 2). These RT and CT were measured at the subfoveal and at 500 μm intervals from the fovea to 3 mm superior, nasal, inferior, and temporal sectors (20).

### Data Analysis

All data were analyzed with an analysis software program (SPSS 18.0; SPSS, Inc., Chicago, IL). All data were reported as mean ± standard deviation (SD), with a 95% confidence interval (CI). Analysis of variance (ANOVA) was used to analyze differences in retinal and choroidal thickness among normal, LM, MM, and HMA. Pearson correlation was used to analyze the relationship between subfoveal RT (SFRT), SFCT, and axial length, and gender. A value of *p* < 0.05 was considered statistically significant.

## Results

A total of 75 Chinese children (128 eyes) were recruited in this study, including 22 NG eyes, 37 LM eyes, 22 MM eyes, 22 HM eyes, and 25 HMA eyes. The mean age of the participants was 10.5 years (range, 4–15 years), and there were 45 males (60%) and 30 females (40%). With reference to RT, no significant differences in subfoveal region were observed between HMA and the other four groups (all *p* > 0.05) (Tables 2, 3). However, at 0.5 mm, RT in the superior, inferior, and temporal sectors were thinner in the HMA patients compared with the LM and MM groups (*p* < 0.05), while no significant differences were observed compared to the NG and HM group (*p* > 0.05). At 1.0 mm, RT was significantly thinner in all four sectors in HMA compared to the LM and MM groups (*p* < 0.05). Meanwhile, compared to the NG, RT was significantly thinner in the inferior, nasal, and temporal sectors (*p* **=** 0.000) in HMA, while no significant differences were observed for the superior sector. In addition, in HMA, RT was thinner in inferior sector compared to the HM group (*p* < 0.05). At 1.5 mm (except in the superior sector) and at 2.0 mm (except in the temporal sector) no significant differences were found between HMA and other groups, and the other measurement points were the thinnest compared with the other four groups (*p* < 0.05). At 2.5 mm, no difference in the RT of the inferior and temporal sectors were found between HM and MM groups (*p* > 0.05). At 3.0mm, HMA showed statistically significant thinning compared with NG, LM, and MM in all four directions (*P* <0.05), and there was also significant thinning compared with HM in inferior(*P* < 0.05).

**Table 2:** Subfoveal retinal and choroidal thickness, axial length in all five groups.

**Groups**	***N***	**Subfoveal retinal thickness** **(mean ±SD) μm**	***P-*value**	**Subfoveal choroid thickness** **(mean ±SD) μm**	***P-*value**	**Axis length** **(mean ±SD) μm**	***P-*value**
High myopia amblyopia	25	218.22 ± 16.89		177.79 ± 64.46		26.70 ± 1.32	
Normal	22	214.72 ± 14.10	0.997	330.20 ± 89.38	0.000	23.04 ± 0.89	0.000
Low myopia	37	222.63 ± 11.48	0.957	305.54 ± 70.75	0.000	24.24 ± 0.71	0.000
Moderate myopia	22	217.93 ± 12.12	1.000	297.68 ± 63.82	0.000	25.02 ± 0.92	0.000
High myopia	22	222.28 ± 15.76	0.996	227.50 ± 66.07	0.164	26.14 ± 1.03	0.788

**Table 3:** Retinal thickness in each group.

**Retinal thickness (mean** **±SD)** **μm**													
**Position**	**Groups**	**0.5 mm**	***P-*** **value**	**1.0 mm**	***P-*** **value**	**1.5 mm**	***P-*** **value**	**2.0 mm**	***P-*** **value**	**2.5 mm**	***P-*** **value**	**3.0 mm**	***P-*** **value**
Superior	High myopia amblyopia	293.58 ± 25.71		322.20 ± 14.24		305.33 ± 13.93		283.29 ± 15.57		265.91 ± 17.04		254.41 ± 17.58	
	Normal	306.31 ± 17.47	0.430	344.77 ± 36.36	0.106	340.54 ± 24.50	0.000	316.72 ± 20.51	0.000	298.90 ± 20.22	0.000	282.13 ± 16.64	0.000
	Low myopia	314.72 ± 15.60	0.009	354.10 ± 11.70	0.000	341.00 ± 13.43	0.000	317.48 ± 12.39	0.000	294.51 ± 13.11	0.000	280.24 ± 12.29	0.000
	Moderate myopia	313.09 ± 12.81	0.023	348.59 ± 14.34	0.000	332.04 ± 17.68	0.000	306.86 ± 17.57	0.000	286.90 ± 14.88	0.001	274.90 ± 14.96	0.001
	High myopia	299.15 ± 14.40	0.991	330.36 ± 20.86	0.815	321.42 ± 22.17	0.095	311.52 ± 28.57	0.006	287.94 ± 21.52	0.009	273.63 ± 24.42	0.067
Nasal	High myopia amblyopia	285.25 ± 22.60		320.95 ± 15.69		315.29 ± 14.26		294.54 ± 15.30		273.66 ± 17.89		263.04 ± 17.45	
	Normal	295.95 ± 21.23	0.669	348.90 ± 18.59	0.000	354.09 ± 24.15	0.000	334.90 ± 26.48	0.000	312.86 ± 25.19	0.000	302.09 ± 25.00	0.000
	Low myopia	300.44 ± 15.55	0.065	351.08 ± 12.01	0.000	351.32 ± 11.28	0.000	331.18 ± 11.99	0.000	309.75 ± 13.14	0.000	296.24 ± 14.66	0.000
	Moderate myopia	300.86 ± 19.56	0.147	344.04 ± 16.13	0.000	343.31 ± 16.71	0.000	325.09 ± 17.65	0.000	304.68 ± 17.34	0.000	292.95 ± 18.09	0.000
	High myopia	285.78 ± 13.13	1.000	331.68 ± 11.38	0.123	335.26 ± 16.27	0.002	318.15 ± 19.76	0.001	296.00 ± 22.24	0.011	278.15 ± 20.99	0.152
Inferior	High myopia amblyopia	288.54 ± 24.17		313.41 ± 19.43		290.25 ± 17.69		262.83 ± 20.06		244.20 ± 19.74		233.00 ± 20.43	
	Normal	303.90 ± 20.15	0.211	349.86 ± 17.26	0.000	330.50 ± 17.26	0.000	304.77 ± 25.96	0.000	278.31 ± 17.24	0.000	267.22 ± 14.76	0.000
	Low myopia	311.24 ± 18.18	0.003	347.54 ± 12.97	0.000	327.81 ± 14.87	0.000	296.59 ± 13.14	0.000	272.24 ± 12.18	0.000	264.78 ± 14.20	0.000
	Moderate myopia	306.40 ± 13.86	0.036	340.63 ± 13.17	0.000	320.45 ± 16.05	0.000	288.00 ± 16.99	0.000	267.36 ± 16.75	0.001	257.54 ± 18.56	0.001
	High myopia	294.05 ± 15.85	0.991	330.63 ± 13.98	0.016	316.26 ± 20.04	0.001	288.21 ± 23.32	0.006	264.84 ± 27.54	0.093	256.05 ± 26.98	0.040
Temporal	High myopia amblyopia	276.41 ± 21.01		308.33 ± 14.46		297.70 ± 13.39		275.75 ± 20.60		251.45 ± 14.75		237.33 ± 13.49	
	Normal	290.18 ± 18.97	0.217	333.81 ± 16.59	0.000	327.13 ± 19.56	0.000	303.72 ± 20.13	0.000	280.59 ± 16.52	0.000	262.68 ± 14.89	0.000
	Low myopia	298.43 ± 15.15	0.001	334.75 ± 11.23	0.000	325.29 ± 11.76	0.000	299.91 ± 12.18	0.000	277.02 ± 10.89	0.000	260.75 ± 9.62	0.000
	Moderate myopia	292.31 ± 13.50	0.037	326.45 ± 14.77	0.001	316.27 ± 17.98	0.003	292.27 ± 18.65	0.064	269.04 ± 18.64	0.011	252.81 ± 16.45	0.012
	High myopia	282.78 ± 10.49	0.897	314.73 ± 25.87	0.985	313.26 ± 16.80	0.023	289.21 ± 18.66	0.266	266.52 ± 18.75	0.069	249.84 ± 19.10	0.197

The CT at the subfoveal and 0.5 mm/1.0 mm/1.5 mm/2.0 mm/2.5 mm/3.0 mm in the four directions (superior/nasal/inferior/temporal) was significantly thinner in HMA compared with NG, LM, and MM groups (*p* = 0.000) (Tables 2, 4). In addition, compared with HM, CT in HMA was also significantly thinner at 1.0 mm/1.5 mm/2.0 mm/2.5 mm/3.0 mm in superior and inferior sectors (*p* < 0.05); nevertheless, there was no significant difference in the nasal and temporal sectors (*p* > 0.05).

**Table 4:** Choroidal thickness in each group.

**Choroidal thickness (mean** **±SD)** **μm**													
**Position**	**Groups**	**0.5 mm**	***P-*** **value**	**1.0 mm**	***P-*** **value**	**1.5 mm**	***P-*** **value**	**2.0 mm**	***P-*** **value**	**2.5 mm**	***P-*** **value**	**3.0 mm**	***P-*** **value**
Superior	High myopia amblyopia	181.29 ± 61.75		181.66 ± 60.29		181.66 ± 60.29		186.56 ± 58.81		191.86 ± 55.97		193.56 ± 54.46	
	Normal	346.77 ± 93.59	0.000	349.04 ± 91.83	0.000	349.04 ± 91.83	0.000	348.95 ± 90.04	0.000	349.59 ± 89.33	0.000	345.13 ± 89.49	0.000
	Low myopia	314.70 ± 73.09	0.000	315.62 ± 70.84	0.000	315.62 ± 70.84	0.000	309.00 ± 68.80	0.000	306.75 ± 71.81	0.000	303.37 ± 73.47	0.000
	Moderate myopia	307.09 ± 64.68	0.000	307.81 ± 65.17	0.000	307.81 ± 65.17	0.000	306.63 ± 66.42	0.000	302.86 ± 68.97	0.000	298.18 ± 70.45	0.000
	High myopia	232.15 ± 60.19	0.093	236.31 ± 55.64	0.037	236.31 ± 55.64	0.038	245.52 ± 59.72	0.027	254.26 ± 60.26	0.014	260.00 ± 61.63	0.008
Nasal	High myopia amblyopia	162.41 ± 70.57		150.54 ± 74.57		126.70 ± 66.96		106.45 ± 57.42		94.54 ± 41.98		85.75 ± 38.63	
	Normal	304.90 ± 89.93	0.000	285.81 ± 94.14	0.000	267.00 ± 95.56	0.000	247.54 ± 95.04	0.000	222.72 ± 91.26	0.000	192.77 ± 82.12	0.000
	Low myopia	280.02 ± 68.30	0.000	259.27 ± 66.46	0.000	234.45 ± 62.57	0.000	210.51 ± 58.72	0.000	189.67 ± 59.30	0.000	161.72 ± 46.96	0.000
	Moderate myopia	275.45 ± 66.95	0.000	257.81 ± 66.34	0.000	236.90 ± 61.63	0.000	212.59 ± 53.23	0.000	187.31 ± 47.24	0.000	160.22 ± 41.69	0.000
	High myopia	208.89 ± 67.52	0.292	192.05 ± 65.15	0.454	173.10 ± 66.09	0.252	155.36 ± 70.82	0.183	137.94 ± 74.14	0.269	125.68 ± 73.15	0.341
Inferior	High myopia amblyopia	180.37 ± 58.84		177.33 ± 59.44		179.00 ± 56.33		181.25 ± 53.18		173.70 ± 50.00		162.12 ± 44.56	
	Normal	331.45 ± 96.49	0.000	325.18 ± 95.76	0.000	317.63 ± 94.35	0.000	308.50 ± 91.62	0.000	300.63 ± 87.66	0.000	290.72 ± 82.51	0.000
	Low myopia	310.05 ± 74.57	0.000	304.86 ± 73.84	0.000	299.56 ± 70.63	0.000	293.70 ± 66.91	0.000	289.05 ± 64.07	0.000	279.59 ± 58.96	0.000
	Moderate myopia	301.59 ± 68.04	0.000	297.45 ± 67.83	0.000	293.95 ± 65.29	0.000	289.54 ± 63.32	0.000	281.72 ± 59.80	0.000	269.72 ± 55.24	0.000
	High myopia	235.57 ± 69.43	0.086	241.47 ± 72.10	0.033	250.31 ± 72.34	0.012	254.73 ± 70.53	0.006	255.36 ± 64.60	0.001	248.63 ± 62.04	0.000
Temporal	High myopia amblyopia	187.83 ± 66.94		194.00 ± 64.38		198.37 ± 59.86		207.00 ± 54.40		210.25 ± 54.90		209.66 ± 56.11	
	Normal	329.13 ± 77.51	0.000	332.86 ± 74.45	0.000	332.77 ± 72.49	0.000	328.77 ± 70.88	0.000	322.45 ± 70.40	0.000	311.59 ± 70.56	0.000
	Low myopia	307.75 ± 68.04	0.000	312.24 ± 69.62	0.000	313.35 ± 69.61	0.000	312.81 ± 66.58	0.000	308.94 ± 67.01	0.000	299.97 ± 66.78	0.000
	Moderate myopia	297.27 ± 59.66	0.000	302.27 ± 55.83	0.000	305.27 ± 55.03	0.000	305.31 ± 53.72	0.000	302.04 ± 50.90	0.000	289.90 ± 44.53	0.000
	High myopia	232.63 ± 67.65	0.311	239.68 ± 66.54	0.256	247.10 ± 60.86	0.117	249.94 ± 60.42	0.189	251.15 ± 61.93	0.262	249.00 ± 63.22	0.336

**Figure 3:**
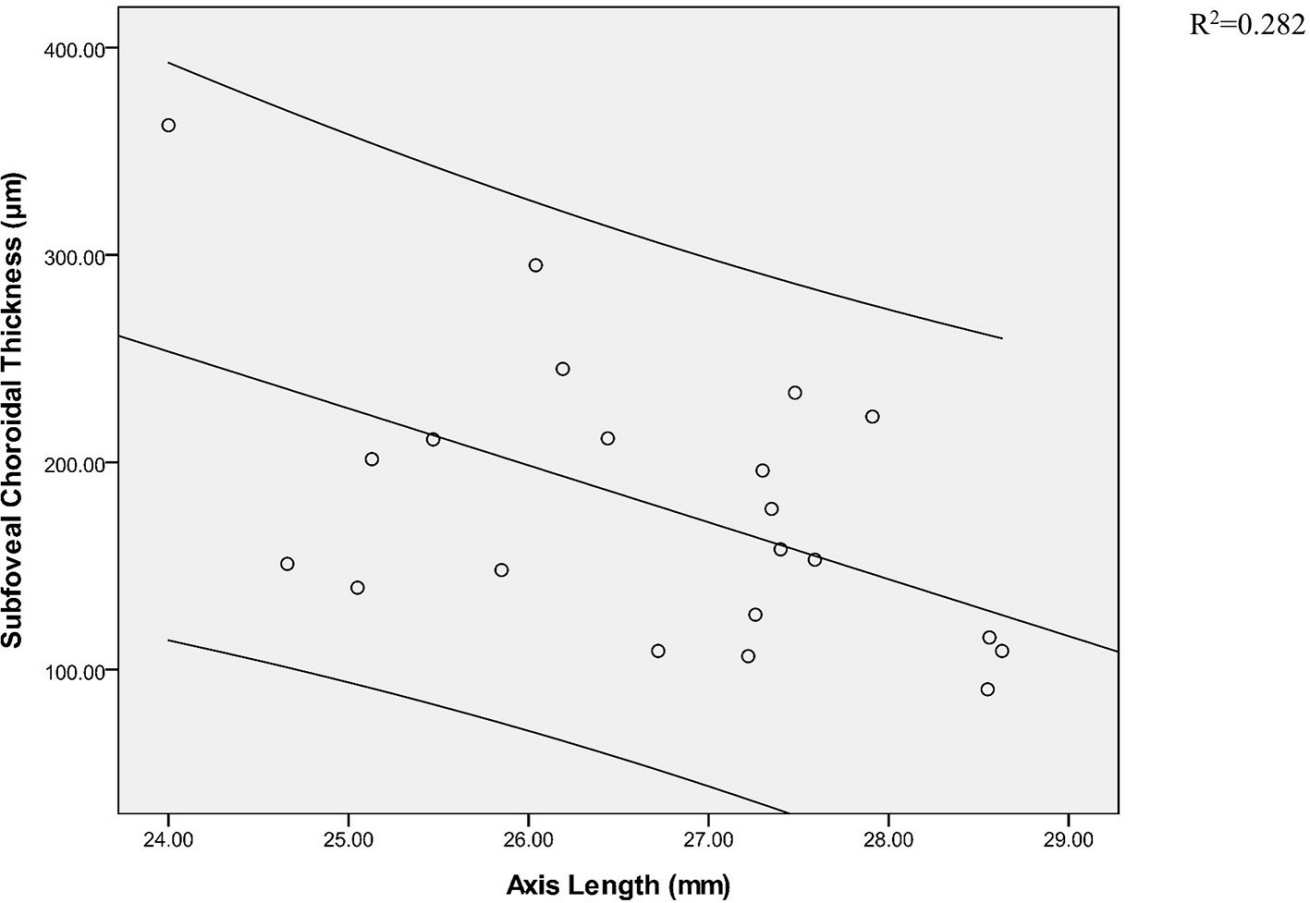
Pearson correlation analysis of subfoveal choroidal thickness and axial axis in high myopic amblyopic eyes.

As shown in Tables 5, 6, in the five groups, SFRT and SFCT were not correlated with age and sex, and there was no significant correlation between SFRT and AL (Table 7). SFCT in HMA, HM, and NG was negatively correlated with AL. In addition, the SFCT in HMA (*r* = −0.531; *p* = 0.013) showed a correlation with AL for the subfoveal location, similar to NG (*r* = −0.538; *p* = 0.010). Compared with LM (*r* = −0.334; *p* = 0.050), MM (*r* = −0.353; *P* = 0.108), and HM (*r* = −0.483; *p* = 0.036), SFCT in HMA (*r* = −0.531; *p* = 0.013) showed a negative correlation with AL (Figure 3, Table 8).

**Table 5:** Correlation between subfovea retinal thickness and age in each group.

**Age**	**Subfoveal retinal thickness**				
	**Normal group**	**Low myopia group**	**Moderate myopia group**	**High myopia group**	**High myopia amblyopia group**
*R*	−0.041	0.352	0.234	0.528	0.220
*R* ^2^	0.002	0.124	0.055	0.278	0.048
*F*	0.034	4.962	1.162	6.555	1.115
*P*	0.856	0.032	0.294	0.020	0.302

**Table 6:** Correlation between subfovea choroidal thickness and age in each group.

**Age**	**Subfoveal choroidal thickness**				
	**Normal group**	**Low myopia group**	**Moderate myopia group**	**High myopia group**	**High myopia amblyopia group**
*R*	0.201	0.094	0.185	0.073	0.292
*R* ^2^	0.040	0.009	0.034	0.005	0.085
*F*	0.841	0.311	0.707	0.091	2.044
*P*	0.370	0.581	0.410	0.766	0.167

**Table 7:** Correlation between subfovea retinal thickness and axial length in each group.

**Axial length**	**Subfoveal retinal thickness**				
	**Normal group**	**Low myopia group**	**Moderate myopia group**	**High myopia group**	**High myopia amblyopia group**
*R*	0.131	0.352	0.114	0.341	0.142
*R* ^2^	0.017	0.124	0.013	0.116	0.020
*F*	0.351	4.680	0.263	2.231	0.392
*P*	0.560	0.038	0.613	0.154	0.539

**Table 8:** Correlation between subfovea choroidal thickness and axial length in each group.

**Axial length**	**Subfoveal choroidal thickness**				
	**Normal group**	**Low myopia group**	**Moderate myopia group**	**High myopia group**	**High myopia amblyopia group**
*R*	−0.538	−0.334	−0.353	−0.483	−0.531
*R* ^2^	0.289	0.111	0.124	0.234	0.282
*F*	8.132	4.140	2.839	5.184	7.476
*P*	0.010	0.050	0.108	0.036	0.013

## Discussion

High myopic amblyopia is a special type of amblyopia that has received little attention. In this study, we used EDI-OCT to evaluate the mean RT and CT in different refractive eyes.

Previous studies have reported that RT showed variations by sex and age, and CT can be significantly influenced by age and AL in normal eyes (21, 22). Nevertheless, in this study, we found no correlation between SF RT/SFCT and age (Tables 5, 6) in NG, as well as those with LM, MM, or HMA. In addition, no significant correlation between SFRT and AL was observed between the groups (Table 7). However, SFCT was negatively correlated with AL in NG, HM, and HMA (Table 8). Furthermore, after examining the RT of 0.5 mm to fovea, HMA was the significantly thinnest compared to LM and MM in three sectors (superior, inferior, and temporal); nevertheless, no significant differences were found compared to HM. In addition, no statistical difference at SFRT was found between HMA and other groups. Our data was not consistent with previous reports. Pang et al. (10) examined 31 amblyopic eyes with HM and found significant changes in macular thickness (MT) between amblyopic eyes and normal fellow eyes. Moreover, similar data were reported by Araki et al. (23), who measured 46 amblyopic eyes (31 with myopic anisometropia and 15 with hyperopic anisometropia). Average MT was calculated in the center, inner, and outer macular regions, and the normal eyes did not have SE grading, which might explain the difference in their results.

Although the area of 1 mm diameter range around the fovea is the main imaging area for central vision, our results suggested that the structure of the retina cannot fully reflect its function, and we should look at the problem as a whole rather than locally. In this study, we found that at 1.0 mm/1.5 mm/2.0 mm/2.5 mm/3.0 mm to fovea, HMA has thinner RT in all four sectors compared to NG, LW, and MM, except for two points. RT of HMA was thinner than HM at each measurement point in the four directions, and was more significant in the inferior, with statistical difference. This may be due to particularity of HMA, in which retinal structure itself is different. It might also be that the retinal tissue structure becomes thinner as the AL increases. According to our current study, there was no difference between HMA and HM on AL, while the treatment effect was poor in HMA. BCVA was below normal, which induced the question whether reducing RT to a certain extent would affect VA.

Yoon et al. (24) and Dickmann et al. (25) have investigated different types of amblyopia and found no difference in MT between the amblyopic and normal fellow eyes. In addition, Park and his team (26) reveal differences between amblyopic and fellow eyes in the thickness of some retinal layers, including a notable difference in the ganglion cell layer plus inner plexiform layer. Although, these studies included subjects with hyperopic amblyopia, whereas in the current study, we examined subjects with myopia and myopic amblyopia. In this study, participants with HMA were compared to HM, spherical equivalent (SE) refractive errors of these eyes was less than −6.0 D and no significant difference in AL was found between these two groups. Nonetheless, RT at most of the measurement points was statistically thinner (especially in the inferior sector) in the HMA compared to HM.

Furthermore, we found that HMA has thinner CT compared to NG, LW, and MM. In addition, at 1.0 mm/1.5 mm/2.0 mm/2.5 mm/3.0 mm in superior and inferior sectors, HMA showed statistically thinner CT compared with HM, while no significant difference between HMA and HM was observed at subfoveal and 1.0 mm/1.5 mm/2.0 mm/2.5 mm/3.0 mm in nasal and temporal sectors. Spaide et al. (11) used EDI technique to examine the SFCT in 17 normal subjects (average age was 33.4 years) and found that SFCT was 318 μm in right eyes and 335 μm in left eyes. Moreover, Margolis and Spaide (20) reported an SFCT of 287 μm in 30 subjects with an average age of 50.4 years, where nasal thickness rapidly decreased, thus reaching a minimum mean of only 145 μm at 3 mm nasal to the fovea. Ding et al. (27) have reported that SFCT was 261.93 μm in 210 volunteers (mean age, 49.73 years). Our present study showed that SFCT was 330.20 μm in 19 normal Chinese children subjects (22 eyes) with an average age of 10.18 ± 2.63 years, 262.68 μm at 3 mm of temporal sector, and 302.09 μm at 3 mm of nasal sector. Our results differed from previous studies in that our subjects were significantly younger than the average age of the study population, and choroid thickness thins with age.

With reference to the hyperopic amblyopi a, a large number of previous articles (28–32) have reported that SFCT with hyperopic anisometropic amblyopia is significantly thicker compared to the fellow eye and the age-matched controls. Yet, only a few studies have suggested that CT is related to the occurrence of amblyopia (29, 31, 32). Xu and colleagues (31) have suggested that SFCT in amblyopic eyes negatively correlated with AL, but did not correlate with SE, VA, or age. However, Xu et al. (31) did not describe the RT. Moreover, Araki et al. (23) found that in the strabismic amblyopia group, there was no significant difference in the mRNFL, GCL+IPL, GCC thicknesses and CT (subfovea, center 1 mm, nasal and inferior of the inner ring, nasal of the outer ring, and center 6 mm) among the amblyopic, fellow, and control eyes."

In this study, obviously thinner CT was observed at 1.0 mm/1.5 mm/2.0 mm/2.5 mm/3.0 mm in the superior and inferior sectors of HMA compared with HM. In addition, the CT was thinner in the inferior sector compared to the superior one. At the same time, at the 0.5 mm area outside the fovea in the inferior sector, HMA had thinner RT compared to HM, while no significant difference in SFCT and SFRT were observed between the two groups. This was a novel finding that was not consistent with previous research. Consequently, our data suggested that CT thinning is associated with RT thinning in HMA, and amblyopia influences RT and CT. Thus, thinner choroid supplies less blood to the outer retina, resulting in thinning of the retina. In our current study, we used different measurements, which is why the obtained data do not adequately reflect the areas. Therefore, future studies should use SD-OCT to measure RT and CT in four directions in HMA.

In the present study, changes in the RT and CT in HMA were the most remarkable in inferior area, except for the 0.5 mm measurement point. However, this still does not explain why HMA occurs.

Previous reports have disputed about CT before and after treatment for amblyopia. Araki et al. (33) have reported that there were no significant changes in SFCT, center 1 mm CT, or center 6 mm CT before and after treatment in the amblyopic and fellow eyes. In addition, Liu et al. (34) have used a meta-analysis and reported that the SFCT in unilateral amblyopia was thicker than that in the fellow and control eyes. In the current study, we did not observe the CT before and after the treatment of HMA, which should be addressed in future studies. On the other hand, Pang and his team (35) have reported that contrast sensitivity function at the middle and higher frequencies was reduced in the amblyopic eyes associated with myopic anisometropia compared to the fellow eyes with high myopia. Therefore, further studies on visual function are also necessary.

This study has the following limitations: First, it is difficult to match HMA and HM in the same subject. If HMA and HM can be identical with different eyes of the subject, the errors caused by individual differences can be excluded. Second, there were fewer subjects in the study. Third, no MRI was performed to rule out visual abnormalities.

## Data Availability Statement

The original contributions presented in the study are included in the article/supplementary material, further inquiries can be directed to the corresponding author.

## Ethics Statement

The studies involving human participants were reviewed and approved by Ethics Committee of Guangzhou Hospital of Integrated Traditional and Western Medicine. Written informed consent to participate in this study was provided by the participants' legal guardian/next of kin.

## Author Contributions

All authors listed have made a substantial, direct, and intellectual contribution to the work and approved it for publication.

## Funding

This study was supported by Guangzhou Huadu District Science and Technology Project of China (14-HDWS-026).

## Conflict of Interest

The authors declare that the research was conducted in the absence of any commercial or financial relationships that could be construed as a potential conflict of interest.

## Publisher's Note

All claims expressed in this article are solely those of the authors and do not necessarily represent those of their affiliated organizations, or those of the publisher, the editors and the reviewers. Any product that may be evaluated in this article, or claim that may be made by its manufacturer, is not guaranteed or endorsed by the publisher.

## Abbreviations

HMA: high myopic amblyopia
LM: low myopia
MM: moderate myopia
HM: high myopia
NG: normal group
SD-OCT: spectral-domain optical coherence tomography
RT: retinal thickness
CT: choroidal thickness
EDI: enhanced depth imaging
AL: axial length
BCVA: best-corrected visual acuity
SF: subfoveal
MT: macular thickness.
